# Challenges in the cross-sectoral collaboration on vulnerable pregnant women: a qualitative study among Danish general practitioners

**DOI:** 10.1186/s12875-022-01773-0

**Published:** 2022-07-26

**Authors:** L. Brygger Venø, L. B. Pedersen, J. Søndergaard, R. K. Ertmann, D. E. Jarbøl

**Affiliations:** 1 Department of Public Health, Research Unit of General Practice, University of Southern Denmark, Odense, Denmark; 2 Department of Public Health, DaCHE - Danish Centre for Health Economics, University of Southern Denmark, Odense, Denmark; 3 Department of Public Health, Research Unit of General Practice, University of Copenhagen, Copenhagen, Denmark

**Keywords:** Vulnerability, Pregnancy, General practice, Cross-sectoral collaboration, Antenatal care, Social reporting, Barriers, Facilitators

## Abstract

**Background:**

Vulnerable pregnant women, defined as women threatened by social, psychological, or physical risk factors, need special support during pregnancy to prevent complications in pregnancy, birth, and childhood. Proper cross-sectoral collaboration in antenatal care is paramount to delivering sufficient supportive care to these women. General practitioners (GPs) often face barriers when assessing vulnerable pregnant women and may; as a result, under-identify and underreport child abuse. Little is known about how the cross-sectoral collaboration in antenatal care affects the GP’s opportunities of managing vulnerable pregnant women. This study explores GPs’ perceived barriers and facilitators in the antenatal care collaboration on vulnerable pregnant women and in the reporting of these women to social services.

**Methods:**

A qualitative study with semi-structured focus group interviews among twenty GPs from the Region of Southern Denmark. A mixed inductive and deductive analytic strategy was applied, structured according to the Theoretical Domains Framework (TDF).

**Results:**

Three themes emerged: I) collaborative experience, II) motivation, and III) organizational working conditions. Barriers were lacking experience, i.e. knowledge, skills, and attention to antenatal care collaboration and reporting, inadequate organizational working contexts, i.e. insufficient pathways for communication between health care and social care systems, and laws restricting feedback on the consequences of reporting. This decreased the GPs motivation, i.e. poor confidence in navigating the system, fear of breaking the patient alliance when collaborating in antenatal care and reporting with the social services. GPs motivation to collaborate and report was increased by knowing the working contexts of their collaborative partners in the antenatal care and social services system and by a strong doctor-patient relationship enabling them to describe the vulnerability to collaborators.

**Conclusions:**

GPs experience system-related barriers to collaborating and reporting on vulnerable pregnant women within the health care sector and in the interplay with the social services sector. Organizational development of cross-sectoral antenatal care collaboration should imply user involvement of all collaborative partners. Results suggest that health authorities should consider establishing accessible communication pathways between the GPs and the social services to improve options for proper cross-sectoral communication and feedback to GPs, thereby improving care trajectories of vulnerable pregnant women.

**Supplementary Information:**

The online version contains supplementary material available at 10.1186/s12875-022-01773-0.

## Background

An increasing number of women of the fertile age report signs of vulnerability due to mental health and social problems [1]. If the vulnerability is missed among pregnant women, the risk of complications during the pregnancy, birth, and the offspring’s childhood increases [2]. Vulnerability in pregnancy has been described in terms of women threatened by social, psychological, or physical risk factors, combined with a lack of adequate coping skills or support [3–11]. The risk factors could be either obvious or vague [12].

The goal of antenatal care (ANC) is to ensure that pregnant women get the necessary level of care concerning obstetric and psycho-social risk factors to prevent possible complications during pregnancy, birth, and early childhood. Special preventive support is dedicated to vulnerable pregnant women, which involves collaborating with several partners in the health care system and social services system [2]. However, there is a deficit in the ANC for vulnerable pregnant women since less than 25% of pregnant women with severe vulnerability are identified in Danish general practices [13]. In the UK, with a similar health care system, approximately 50% of pregnant women with perinatal depression and anxiety are identified by health care professionals [14]. Studies from other developed countries point out that health care professionals face many barriers when assessing vulnerability among pregnant women [12, 15–19] and that general practitioners (GPs) generally under-identify and underreport child abuse [20–22].

Little is known about how the cross-sectoral collaboration in ANC affects the GP’s opportunity of managing vulnerable pregnant women. Recently, the Royal College of General Practitioners in the UK concluded that inconsistent teamwork between GPs and the cross-sectoral partners in ANC is the most critical barrier to proper collaboration on pregnant women with mental health problems [14]. Experts in health innovation argued that effective changes to the health care system should be based on knowledge of the clinician’s perceived complexity of collaborating in the system [23]. Our pre-assumption in the field derives from our experiences as GPs engaging in the cross-sectoral ANC collaboration on vulnerable pregnant women. We assume that GPs are influenced by multiple barriers such as insufficient communication pathways and lacking cross-sectoral transmission of relevant information indicating vulnerability in families. We had experienced how vulnerable women had sometimes slipped through our hands since important information indicating vulnerability in our patients existed in the social system, i.e., social support needs. This information is only shared coincidentally through the patient. We believe that the lacking cross-sectoral transmission of information on vulnerability indicators can limit the GPs’ attention on vulnerability in pregnancy. This article aims to explore GPs’ perceived barriers and facilitators in the cross-sectoral collaboration on the care of vulnerable pregnant women and in reporting on these women to the social services.

## Methods

### Design and theoretical framework

The study is a qualitative interview study based on a focus group method approach with semi-structured discussions among GPs. It is part of a multi-method project exploring barriers and facilitators for assessing and managing ANC of vulnerable pregnant women in general practice [12, 24]. The research group developed the interview guide based on a literature search, experience working with ANC, field studies in social-obstetric units, interviews with social workers and health visitors, and a pilot focus group interview with GPs. The interview guide (Additional file 1: appendix 1) comprised open questions on challenges in the cross-sectoral collaboration on vulnerable pregnant women and barriers and facilitators for reporting suspected social support needs of the woman. We chose the focus group method approach with discussions among peers to enable GPs to exchange experiences and inspire each other to reflect on their practices of collaborating and reporting on vulnerable pregnant women. The safe environments rendered a deep nuanced dialogue and encouraged them to disclose deficient performances. This allowed us to achieve deep nuanced insights into the field. The interviewer acted neutral and like-minded without disclosing preconceptions in the area. The study design followed the COREQ criteria [25]. See the complete checklist in Additional file 2: Appendix 2.

As a theoretical framework, we used the Theoretical Domains Framework (TDF), which is based on theories of behavior change. We chose the TDF as it has been shown as an effective theoretical lens to view the GPs’ cognitive, affective, social, and environmental influences in various settings [26–31]. It was developed to identify causes of implementation difficulties and promote understanding of how to change health professionals’ behavior. TDF is based on the integration of 33 theories and 84 constructs from behavioral theory, resulting in 14 theoretical domains useful for categorizing barriers and facilitators to specific behaviors [26, 32–34]. The domains appear in Table 3. TDF has been validated to facilitate research into implementation problems [34, 35], and it has been used in empirical studies addressing GPs’ barriers to implementation problems in different clinical areas [28–31, 36–38]. Therefore, this method was perceived helpful in exploring the GPs’ perceived barriers and facilitators for collaborating and reporting in the cross-sectoral ANC. We developed a codebook accommodating the TDF domains for the concept of cross-sectoral collaboration (See Additional file 3: appendix 3).

### Institutional setting and organization of Danish antenatal care

The study was conducted in a general practice setting in the Region of Southern Denmark. The Danish health care system is free of charge. Almost all Danish citizens are listed with a GP, and approximately 90% of the population visits their GP a least once a year. Patients have a free choice of GP if access is available [39].

**Fig. 1:**
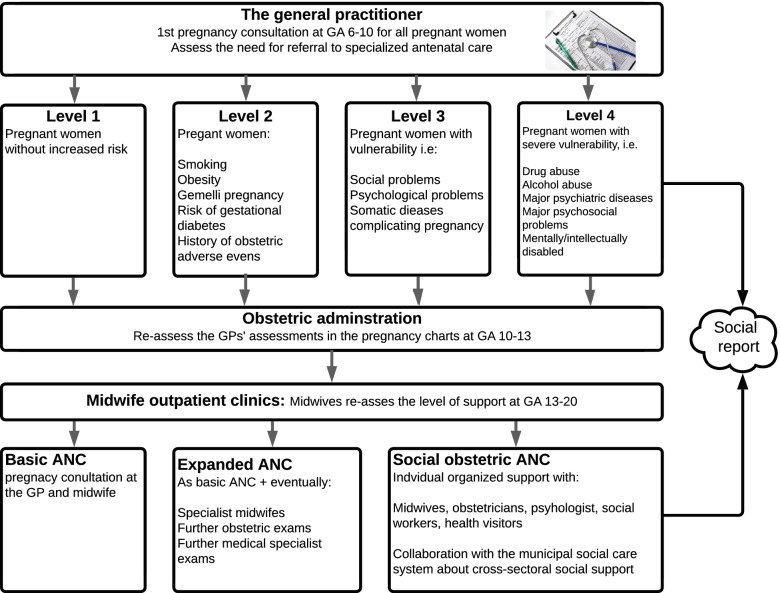
The assessment of pregnant women in Danish antenatal care (ANC). The assessment is dynamic and can be changed depending on events in pregnancy. GPs = general practitioners, GA = gestational age

The Danish ANC system consists of a formalized collaboration between GPs, midwives, obstetric departments and health visitors [2]. All pregnant women consult their GP for a first pregnancy exam at gestational age 6–10, which precedes all other ANC contacts within the health care system. Two additional pregnancy exams are offered in general practice at gestational ages 25 and 32, and a fourth postnatal exam at eight weeks postpartum [2]. The GPs’ task is to assess the woman’s comorbidities and psychosocial resources and decide on referral in one of four levels of ANC. The pregnancy chart functions as a communication and assessment tool, from the GPs to the midwives and the obstetric department, to decide the level of ANC (Fig. 1) [2].

All health care professionals are obliged by law to report suspicions of a severe vulnerability in a pregnant woman or her partner to the social services. This covers the suspicion that a child might need special support immediately after birth due to the parents’ condition [2, 40]. Therefore, referral to level four in ANC mainly implies that the GPs’ should report to the social services [41].

A social report prompts the social services to assess the parenting resources of the pregnant women and their partners and the potential need for social support for families and children [42]. The health care and social services sectors act under separate laws, which hinder the free transmission of information between different sectors [42–44].

### Data collection

Twenty GPs participated in five focus group interviews between March 2019 and January 2020. On average, four GPs participated in each group. Participants’ demographic details are shown in Table 1. The study used purposive sampling to include GPs representative concerning gender, seniority, practice type, and practicing in rural and urban communities with patients from different socioeconomic levels. Recruitment channels were letters, e-mails, and phone calls. GP trainees were included as ANC consultations are conducted by GPs with all seniority ranges.

**Table 1:** Participant demographic details

Years of experience	Practice type	Practice area	Gender
0 years (GP trainees) (3)	Single-handed practices (0)	Urban area (5)	Female (12)
1–5 years (5)	Partnership practices (20)	Semi-urban area (11)	Male (8)
6–10 years (2)		Rural area (4)	
11–15 years (5)			
> 15 years (5)			

The interviews were led by LBV and DEJ and lasted 60 min. The flexible interview guide encouraged free discussion involving all participants. Ongoing adjustments to the interview guide were made to elaborate on newly emerged perceptions of barriers and facilitators. Sampling continued until enough data had been obtained to answer the research question, thereby reaching high information power [45].

### Data management and analysis

All interviews were audio-recorded, transcribed verbatim by LBV and uploaded to NVIVO for analysis. All members of the research group read the first transcripts thoroughly before coding. The data analysis switched between open inductive coding with systematic text condensation [46] and deductive thematic coding to TDF [34], as illustrated in Table 2.

**Table 2:**
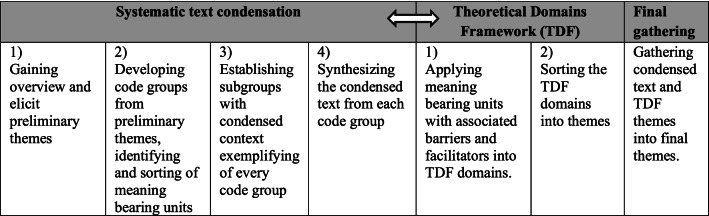
The steps and content of systematic text condensation and TDF

The inductive coding ensured in-depth investigation of themes and subthemes which could emerge freely. The additional focused coding to TDF provided comprehensive coverage of barriers and facilitators. LBV and RE conducted the inductive coding, and JVL assisted the deductive coding by LBV.

**Fig. 2:**
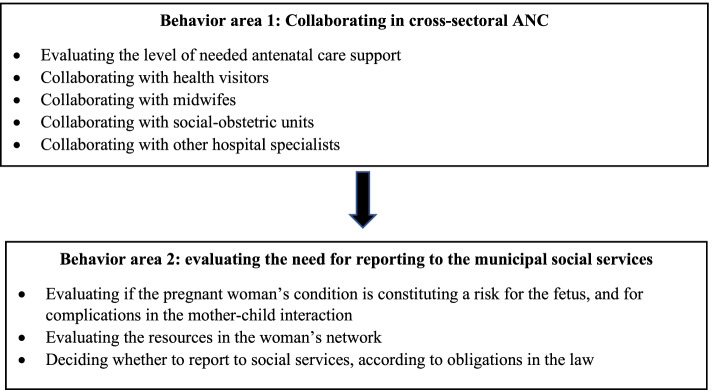
Behavior areas for general practitioners: collaborating in antenatal care (ANC) and reporting to municipal authorities around vulnerable pregnant women

As the standard for managing collaboration around vulnerable pregnant women, we used the Danish guideline recommendations for ANC [2] and the Danish social service law about health care professionals’ obliged duty to report [40]. Inspired by these recommendations, two behavior areas were formulated to guide the analysis (Fig. 2).

## Results

The GPs reported several barriers to accomplishing proper ANC for vulnerable pregnant women related to the cross-sectoral collaboration. Facilitators were also reported, although less prominent. We divided the GPs’ statements according to the two behavioral areas 1) collaboration and 2) reporting to the social services. Three overriding themes emerged with associated barriers and facilitators as shown in Table 3: I) collaborative experience, II) motivation, and III) organizational working conditions.

**Table 3:** GPs’ perceived barriers and facilitators for collaborating and reporting

Behavioral domains	Behavior area 1: Collaborating in cross-sectoral antenatal care (ANC) and social services (SS)		Behavior area 2: Reporting to social services (SS)	
**TDF domains**	**Barriers**	**Facilitators**	**Barriers**	**Facilitators**
***Theme I: Collaborative experience***				
*a. Knowledge*	Uncertainty of content of collaborative pathways in ANC	Knowing the working contexts of collaborative partners in ANC and SS	Lacking knowledge of the rationale behind individual responsibility to report	
*b. Skills*	Lacking experience in navigating ANC and SS system		Lacking trained skills and routine in reporting on vulnerable pregnant women	
*c. Memory, attention and decision processes*	Lacking attention to the benefit of collaborating with ANC and SS professionals	Remembering to collaborate with other ANC and SS professionals	Cognitive limitations, delegating the decision of reporting to other HCPs	Being attentive to the individual responsibility to report
*d. Behavioral regulations*		Efforts to arrange local education on collaboration in the ANC and SS system		
***Theme II: Motivation***				
*e. Social/professional role and identity*		Judged meaningful tasks for GPs	Ethical dilemmas, managing the interests of both the unborn child and the pregnant woman	Keeping the professional obligations in mind
*f. Beliefs about capability*	Low professional confidence navigating in ANC for women with vague indicators of vulnerability	Having confidence in collaborating with vulnerable pregnant women due to existing doctor-patient relation	Low confidence in judging the need for a social report in vulnerable pregnant women	Having confidence in judging the need for reporting obvious threats to the fetus Having a strong doctor-patient relationship
*g. Beliefs about consequences*	Feeling the loss of control when referring to social workers i.n SS		Fear of breaking the doctor-patient alliance	
*h. Optimism*			Pessimism: perceiving that a social report will have no consequence	Optimism: having positive experiences of the consequences of collaborating with SS
*i. Reinforcement*		Remuneration for meetings with municipal social workers and health visitors in SS		
*j. Goal*	Not prioritizing time for collaboration on vulnerable pregnant women			
*k. Intention*	*No coding*			
*l. Emotion*				
***Theme III: Organizational working conditions***				
*m. Environmental context and resources*	Lacking clear and easy communication pathways across sectors Big social services organizations limit collaboration No information on initiatives of social support from SS	Minor size social service organ eases the cross-sectoral collaboration Face-to-face meetings with social workers regarding vulnerable pregnant women	Lacking two-way correspondence systems Lacking feedback from SS on the consequences of a social report	
*n. Social influences*	Dissatisfacting experiences of collaboration lead to a lack of trust in SS	Perceiving positive values of collaborative partners in ANC	Social pressure from SS or other ANC partners to report	Patients wishing for social support

TDF domains (italics in the left column, labelled a-n) are categorized in themes (I-III). Empty boxes mean no barriers/facilitators were identified in the data material. *ANC* antenatal care, *SS* social services

### Behavior area 1: collaborating in the cross-sectional antenatal care

#### Theme I: collaborative experience

The GPs’ expressed varying *knowledge (a)* of the supportive opportunities in ANC for vulnerable pregnant women. Barriers to collaboration were insufficient *knowledge (a)* of the contents of levels in ANC and the working functions of the collaborative partners in ANC, as well as lack of *skills (b)* navigating the system.

*“There’s so many opportunities combined in different ways, so to orient in this jungle… how do you get the pregnant woman or her family to the right opportunity of care? This can be a challenge”* (GP1C, female,< 45 years)

A barrier was that some GPs seemed to lack *attention (c)* to the benefit of collaborating with relevant partners in ANC. Contrary, GPs’ who were attentive to collaborating with relevant ANC partners perceived it to increase the care of vulnerable pregnant women. Some GPs described how their close collaboration with municipal health visitors hindered vulnerable pregnant women from being lost in the system.

A GP realized the need for increased knowledge of the interplay between the different partners in ANC. This led to *behavioral regulations (d)* with efforts to arrange local education in the clinic, which increased their capability to collaborate more efficient in ANC.

#### Theme II: motivation

The GPs’ agreed that collaborating in ANC on vulnerable pregnant women was a meaningful part of their *social and professional role (e).*

Motivational barriers to collaboration on vulnerable pregnant women were related to low professional confidence. The GPs were confident navigating in ANC for the women with well-known obvious psychiatric diseases or social problems. However, they had low *beliefs in their capabilities (f)* navigating in the ANC collaboration on pregnant women, when the vulnerability indicators were rather vague, and their concern was based on their gut feeling.

*“I have this pregnant girl, well-educated with a history of having a kind of personality disorder (…). I can’t give her a diagnosis. I can’t send her to the social-obstetric clinic – she’s too old for that, but I have this feeling that she does not have the eligibility to be a parent (…). I feel like I don’t have any options to act… I simply don’t know who to communicate with”.* (GP3C, female, > 45 years).

GPs reporting to have a strong relationship with their vulnerable pregnant patients were more confident and *believed in their capabilities (f)* of collaborating in ANC.

“*There are women I call fragile, with whom I’m having a great relationship and will consult frequently in my attempt to make a great socio-medical effort. Meanwhile, I would put effort into describing my acquaintance of her personality and her family – leaving it for the obstetricians to decide the level of support*” (GP2B, female, > 45 years)

Some GPs reported experiencing a loss of control while collaborating with the social services and *believed the consequences (g)* that involving the social services could break their alliance with the pregnant woman. This was due to fear of complaints about circumventing confidentiality or that the patient would move to another GP.

The GPs were aware of the collective agreement *reinforcing (i)* collaboration with ANC and social services partners by financially remunerating participation in cross-sectoral meetings. However, the GPs’ reported varying attitudes on the benefit of participating in these meetings and whether the remuneration counterbalanced the time spent.

The GPs expressed varying *goals (j)* regarding whether to prioritize cross-sectoral collaboration on their vulnerable pregnant patients, both regarding time spent on telephone calls and participating in cross-sectoral collaborative meetings outside their clinics. Some GPs prioritized participating in cross-sectoral meetings with the social-obstetric units or social services, whereas others reluctantly argued the time missed for other patients. As a GP prioritized on his pregnant patient with schizophrenia:

“*The social-obstetric unit arranged a network meeting with many collaborative partners where I was invited too, but I chose not to participate for several reasons. It was placed at a foolish time during noon, and I would have to cancel my whole noon schedule. So, I agreed with the patient that she could brief me afterwards*.” (GP5A, male, > 45 years)

#### Theme III: organizational working conditions

The GPs perceived their *environmental context and resources (m)* as barriers to ANC collaboration due to troublesome pathways of cross-sectoral communication. Especially lack of clear and easy communication pathways with social workers in the social services was perceived as a collaborative barrier. The GPs’ reported difficulties navigating the extensive sized social services and troubles finding the relevant social worker to discuss minor concerns of vulnerability. They believed that two-way electronic communication was unavailable between the health care sector and social services regarding minor concerns of vulnerability. Their only option to communicate with the social services was through a social report, which was perceived as exaggerated in situations of vague vulnerability based on intuition.

“*In the good old days, we could call the social worker in the municipality for a talk – but we can’t anymore. I can’t remember writing an ordinary letter of concern – I don’t know if it is possible anymore. I think a social report is the only way nowadays (…). We need a communication channel that is more than a social report- versus no social report. Like a two-way communication where you can discuss lower levels of concern”* (GP5A, male, > 45 years)

However, some GPs perceived a better utilization of the cross-sectoral collaboration when practicing in minor municipalities. As a GP stated on collaboration with social services:

“*One or two social workers came to our clinic at scheduled appointments, where we discussed cases of vulnerable pregnant women and their families. Then you got a sense of the different options, and you could discuss things without making a big case out of it.*” (GP1C, female, < 45 years)

Some GPs stated that mutual communication was used insufficiently between health care partners in ANC, leaving information to be delivered through their patients.

*“The midwives and health visitors – they’re running autonomous – You feel that it’s a coincidence what information comes to you. Suddenly a pregnant woman consults you about sleep disturbances, and during the conversation, the woman says that the health visitor is worried about antenatal depression. That information was relevant to me… I miss this dialogue”* (GP1C, female. < 45 years)

Another communicative barrier related to the *environmental context (m)* of cross-sectoral collaboration was that important information on the social resources of a woman or her partner is not shared between the health care sector and the social services sector. A GP reported not being informed about social support initiated by the social services to his female patient, which indicated poor social resources in the family.

“*I remember examples of not being informed about social reports about my patients made by other professionals. I had a family where I was worried and made a social report. Then the mother told me she already had a municipal supportive person in her house, and apparently, a lot of support was initiated that I did not have a clue about. The mother hasn’t told me because she was embarrassed. I would have liked to be informed about such things*” (GP5A, male, > 45 years)

The GPs’ attitudes toward cross-sectoral partners were affected by *social influences (n)*, e.g. socially comparing their values relative to others or alienating experiences leading to a lack of interprofessional trust. Dissatisfying collaboration experiences affected the GP’s value perception and trust in collaborative partners. Especially the GPs’ expressed negative value perceptions of the social workers in social services. Contrary, the GPs’ perceived the health visitors and social-obstetric collaborators in ANC as good-hearted caretaking professionals with the ability to see the women’s needs.

“*I think there is a difference in culture, like social workers, they might be brought up with box-thinking – where should the money come from… It is more the cold part, and then there are the health visitors, who with a good heart can see that there is a pregnant woman or mother who needs extra care*” (GP1A, female, > 45 years)

The GPs’ reported both positive and negative experiences of collaboration with social workers in the social services, where negative experiences were related to situations where their reaction was perceived as exaggerated. This made the GPs reluctant to collaborate with the social services regarding minor concerns of vulnerability in pregnant women. A GP told how she perceived a pregnant woman with a dependent personality disorder as vulnerable due to loneliness. The GP offered the woman conversational therapy in practice. Meanwhile, she reported to the social services with the intention that the woman would get further social support. But the GP was surprised:

*“After having referred this pregnant woman to the social services family department, I was thinking “, what have I exposed this woman for?” because they were going on with all this…she had the feeling that they were evaluating the need for the forcible removal of her child, where I did not doubt her parenting skills. It was totally out of my hands- it was far too violent”* (GP1A, female, > 45 years)

### Behavior area 2: Reporting to social services

#### Theme I: collaborative experiences

Some GPs lacked *knowledge (a)* of the rationality that reporting is an individual responsibility, ensuring municipal support in case the vulnerable pregnant women fail to seek help. Some GPs felt unable to decide upon the need for reporting and delegated this decision to collaboratives with no preexisting familiarity with the woman’s resources (*decision process (c))*. However, other GPs were aware of their obliged duty to report. GPs who delegated the decision to report argued with lack of trained *skills (b)* in reporting about vulnerable pregnant women.

“*It happens so rarely, and therefore I don’t have any routine. There are the social-obstetric units you can refer to, and then they can decide whether to report*”. (GP2F, male GP > 45 years)

A GP whished further *knowledge (a)* of how the social services manage social reports about vulnerable pregnant women.

“*I would like to know how they [the SS] assess these vulnerable pregnant women. Do they differentiate in groups?* “(GP1A, female,> 45 years)

#### Theme II: motivation

The GPs mentioned several barriers decreasing their motivations to report on vulnerable pregnant women. Prominent barriers were ethical challenges in the GPs’ *professional role (e)* related to the duality of handling the interests of both the unborn child with obligations to report and the interest of the pregnant woman.

*“A decision of reporting to social authorities is not a quick decision – it’s weighing the pros and cons of the expected result, how to formulate it so you can argue to the coming mother (..) we have to be able to stand by our decision to report. A report to the social services is an instrument that requires that you’re using your professionalism”* (GP3B, male, > 45 years)

Another de-motivating factor was low confidence in judging the necessity of reporting on vulnerable pregnant women. The GPs were confident in reporting conditions of the pregnant women comprising an apparent threat to the unborn child – i.e. alcohol- or drug abuse during pregnancy. However, most GPs had no or only minor experiences reporting on vulnerable pregnant women. Therefore, they had low *beliefs in their capabilities (f)* judging the necessity of reporting on a vulnerable pregnant woman.

*“If it’s about abuse, then it’s clear, then you’re obliged to report on it, but if it’s just a level of concern – then it’s something else”* (GP2E, female < 45 years old)

A strong doctor-patient relationship could be a barrier to reporting. The GPs described having refrained from reporting due to *beliefs in consequences (g)* of damaging the doctor-patient alliance.

“*It is difficult when we are saying – okay, we are writing a social report and starts up the whole battery. Because then you’re sure that she will not consult you the next time*” (GP3A, female, < 45 years).

Another barrier to reporting was that the GPs’ acquaintances of the situation are often based on diverging histories from the mother and father. The GPs *believed the consequence (g)* that a report could cause families to break up during the pregnancy, thereby damaging the doctor-patient alliance.

However, a strong and trustful doctor-patient relationship was also perceived to increase the GPs’ *beliefs in their capability (f)* arguing the need for support to the pregnant woman, thereby facilitating the decision to report.

A GP expressed a pessimistic attitude belonging to the domain of *optimism (h*) that a social report was sometimes pointless.

*“We might be biased that our family departments in the social services are on their heels and that it will take much before they intervene. (…). If the reports are just lying there collecting dust”* (GP5A, male, < 45 years)

Contrary, GPs’ having positive experiences giving *optimism (h)* about the consequence of collaboration with the social services about vulnerable pregnant women seemed to facilitate the decision to report.

#### Theme III: organizational working conditions

The *environmental contexts(m)* with lack of pathways for two-way correspondence with social services about lower levels of concerns were an additional barrier affecting the GP’s motivation for reporting.

*“So, you’re like in doubt of when to push the button and make the report. Maybe we’re doing all too few reporting, but we’re missing an option for communicating on those who just need a little help without digging down in the deep psychosocial whole”* (GP5A, male, > 45 years)

The GPs were frustrated because they missed feedback from the social services on whether their reports led to any supportive initiatives. They were aware of the obligation of the social services to maintain confidentiality. However, in the context of ANC of vulnerable pregnant women, the concern of confidentiality of the woman was perceived as a barrier to collaboration.

“*I know that the social services are working on the case with my patient, but I don’t know what it is about. It is a huge challenge that their work is surrounded by such secrecy to us, even if the women give consent. It’s my impression that we don’t get any feedback (..) I would like to know which initiatives they put through… e.g. if they put on a supportive person in such a family. All I know is what the woman told me*”. (GP5A, male,> 45 years)

*Social influences (n)* from collaborative partners requesting the GPs to report were a barrier. It brought the GPs into a dilemma between maintaining the interests of the pregnant woman or the care of the coming child.

“*It can be a minefield when a person from the hospital or social services contacts you and ask you to make a social report on a patient where you don’t have enough information to do it. You are not just obliged to report due to the law, but you’re also morally obligated to be properly informed if you’re writing a report*.” (GP3B, male, > 45 years)

Contrary, when the patients themselves wished for help, it was perceived to facilitate reporting. However, this was mostly the case after childbirth and not during pregnancy.

## Discussion

### Statement of principal finding

Several barriers and facilitators related to almost all TDF domains affected the GPs in the cross-sectoral collaboration and reporting on vulnerable pregnant women.

Frequent barriers were associated with lacking interplay between the GPs and health care professionals within ANC and the social services system. Organizational barriers were insufficient mutual communication pathways across sectors and law restrictions limiting feedback on the consequences of a report. This affected the GPs’ motivation -i.e. low confidence in navigating ANC and judging the necessity for reporting, losing control of care trajectories and fear of breaking the doctor-patient alliance.

Facilitators were knowing the working contexts of partners in the ANC and social services and having experience of collaborating and reporting when necessary. This increased the GP’s confidence and motivated collaboration and reporting. Other facilitators were organizational conditions enabling collaborative meetings with partners in the ANC and social services.

### Strengths and weaknesses

The qualitative approach with the collaborative nature of focus group interviews resulted in rich data on GPs’ perceived barriers and facilitators on the subject. Ongoing adjustments were made in the interview guide to elaborate on new perceptions, ensuring coverage of all barriers and facilitators of collaboration and reporting. The study sought to achieve high information power by continuing interviews until reaching a study sample large enough to answer the research question [45]. The COREQ criteria ensured transparency in reflexivity, design, analysis, and findings [25] (appendix 2).

We aimed for a purposeful sample but accepted a convenience sample due to recruitment problems. Of the 60 invited, 20 GPs accepted with lack of time as the main reason to decline. The small-sized focus groups with 3–6 respondents may have limited the broadness of the discussions. However, it could also contribute to respondents feeling of confidentiality and safe space in being able to get themselves heard. Only GPs from partnership practices accepted the invitation, and these could represent the GPs with a particular interest in the subject or time to participate. However, the sample composition varied concerning gender, seniority, and practice location from different sociodemographic areas throughout Southern Denmark. Also, participants differed in frequencies of having ANC consultations indicating that participants were not only the GPs with a particular interest in the subject. We only recruited GPs from one region. The proportion of vulnerable pregnant women may vary slightly between regions, and likewise, the organization of social-obstetric units may vary. However, due to the comprehensive sample composition of GPs, we believe that our findings represent an accurate picture of GPs’ challenges in ANC and therefore are transferable to GPs from other Danish regions.

Using TDF as a theoretical frame gave comprehensive coverage of GPs’ perceived barriers and facilitators in the behavior of collaborating and reporting in the cross-sectoral ANC. Some text may fit multiple TDF domains; nevertheless, it must be coded into the domain which best reflects the key theme [26]. We found meaning bearing units matching 12 out of 14 TDF domains, corresponding with other qualitative studies based on the TDF [27–32, 37]. Overlaps are inevitable since reporting is part of the cross-sectoral collaboration. Yet, reporting was analyzed separately as it reflects more severe management of vulnerability in pregnancy.

The study only reflects the GPs’ perspectives on the collaborative challenges in ANC, which per se must be dynamic. The focus group dynamics might have affected the GPs’ to withhold diverging attitudes or contrary enhanced their sharing of experience. However, we believe that the GPs’ responses reflects individual attitudes.

The research group of GPs possessing years of experience working with ANC in general practice and collaborating with partners in the ANC and social services system gave a good background knowledge of the working conditions and possible challenges for the GPs. On the other hand, our experiences and preconceptions might have affected the generation of the interview guide and the interpretation of the qualitative data. The research team continuously reflected on the contrasts and similarities between the findings and our preconceptions. Inclusion of other professional expertise, e.g. nursing science, midwifery science, or sociology, might have found different perspectives of barriers and facilitators.

### Findings in relation to other studies

#### System-level barriers to collaboration

Similar studies from the UK, Ireland and Australia found system-level barriers relating to poor joint communication between GPs, midwives and health visitors [15, 16, 18]. Even though the organization of ANC might differ between countries, we anticipate that the barriers are comparable. This study adds to the literature in two ways. First, it focuses on GPs’ challenges of collaborating on vulnerable pregnant women as a broader perspective that perinatal mental health problems, as vulnerability also includes social problems and other mental health problems of the woman and her family. Second, it focuses on GP’s views of collaborative challenges within the ANC system as part of the health care sector and between the health care sector and the social services sector.

Our findings reflect how the cross-sectoral collaboration suffers from insufficient possibilities for mutual communication, especially between the GP and the social services sector. In Denmark, reasonable communication pathways exist between collaboratives in the health care system – i.e., between the GPs, hospital outpatient clinics, private medical specialists and healthcare nurses. However, the health care system is separate from the social services system, and the two systems have no tradition of exchanging information on individual patients -e.g. information indicating social vulnerability. Therefore, necessary knowledge is often withheld from the GPs, which otherwise might assist GPs in assessing vulnerability in pregnant women, collaborating in ANC, and deciding upon the need for social reporting.

#### Internal barriers to collaboration

Prioritizing time for cross-sectoral collaboration on vulnerable women was a diverging issue regardless of reinforcing remuneration from collective agreements. Some GPs prioritized time for communication and participation in cross-sectoral meetings with partners in ANC and social services, whereas others would instead prioritize time on other patient categories. These results are supported by studies showing the necessity of allocating time to implement cross-sectoral meetings [47] and that practicing in smaller municipalities or having short or average list sizes predicts GP participation in cross-sectoral meetings [48, 49].

#### Barriers to reporting

The present study shows GPs’ barriers to reporting on vulnerable pregnant women—i.e. lack of training in deciding when to report, lack of trust in social services, and fear of damaging the doctor-patient relationship when reporting. This is consistent with similar findings on GPs’ barriers to reporting child abuse- i.e. lack of time, insecurity in suspicion of child abuse, lack of available support on the decision to report, bad experiences with the social services and fear of damaging the patient-relation [20, 21, 50]. The GPs seem to struggle between maintaining professionalism when balancing the interest of the unborn child versus keeping a positive doctor-patient relationship by managing their patient's interests. While GPs are confident in reporting on obvious signs of vulnerability in pregnancy – i.e. psychiatric disease or severe social problems [12], then the GPs’ professionalism is challenged in the grey zone areas of minor degrees of vulnerability, where no specific guidance exists of either collaborative opportunities or necessities of reporting [12].

## Conclusion

In conclusion, despite a structured and formalized Danish ANC system, there are many challenges in the cross-sectoral collaboration on vulnerable pregnant women both within the health care professionals in ANC and between the healthcare sector and the social services sector. Especially, the GPs were insecure about when to report and found the reporting system inadequate.

### Meaning and implications

An important finding from this study is the need for commissioners to evaluate the possibility of establishing electronic two-way communication pathways between the GPs and the social services sector. This would enable better conditions for cross-sectoral communication and feedback regarding GP’s minor concerns of vulnerability in pregnant women not fulfilling the criteria for a social report. Politicians should also consider ensuring that municipal social services (assuming patient consent) provide sufficient information to GPs about the follow-up taken by the municipality as a consequence of a social report. This is important information that can indicate a potentially vulnerable woman or family. The TDF aims to identify domains applicable in designing interventions [26, 51], and the findings can possibly contribute to future intervention strategies optimizing ANC for vulnerable pregnant women. A step might be a minor scale municipal intervention with user involvement of all collaboratives in ANC and the social services, where both legal opportunities, organizational structures, and remuneration could underpin a better cross-sectoral collaboration.

Continuous education might be necessary to increase the GP’s awareness of the need to report on vulnerable pregnant women and how social services manage social reports.

Studies are needed to elaborate further on the organizational and motivational influences limiting GPs in collaborating and reporting.

## Supplementary Information

**Additional file 1: Appendix 1. **Interview guide.

**Additional file 2: Appendix 2. **COREQ items.

**Additional file 3: Appendix 3. **Theoretical Domains Framework (TDF), domains and constructs coding manual.

## Data Availability

Data supporting the findings of this study were used under a license granted specifically for the current study and therefore is not publicly available according to the data protection regulations from the Danish Data Protection Agency.

## References

[1] Sundhedsstyrelsen [Danish National Board of Health]. Danskernes sundhed - Den Nationale Sundhedsprofil 2021 [The health of the Danes - The National Healthprofile 2021] [Electronic report]. https://www.sst.dk/da/Udgivelser/2022/Danskernes-sundhed: Rosendahls A/S; 2022, March 10th.

[2] Sundhedsstyrelsen [Danish National Board of Health]. Anbefalinger for svangreomsorgen [Recommandatins for antenatal care] 2021. https://www.sst.dk/da/udgivelser/2021/anbefalinger-for-svangreomsorgen2021 [cited 2021.

[3] Beck CT (2001). Predictors of postpartum depression: an update. Nurs Res.

[4] Biaggi A, Conroy S, Pawlby S, Pariante CM (2016). Identifying the women at risk of antenatal anxiety and depression: A systematic review. J Affect Disord.

[5] Kettunen P, Hintikka J (2017). Psychosocial risk factors and treatment of new onset and recurrent depression during the post-partum period. Nord J Psychiatry.

[6] Lancaster CA, Gold KJ, Flynn HA, Yoo H, Marcus SM, Davis MM (2010). Risk factors for depressive symptoms during pregnancy: a systematic review. Am J Obstet Gynecol.

[7] Martini J, Petzoldt J, Einsle F, Beesdo-Baum K, Hofler M, Wittchen HU (2015). Risk factors and course patterns of anxiety and depressive disorders during pregnancy and after delivery: a prospective-longitudinal study. J Affect Disord.

[8] Nielsen Forman D, Videbech P, Hedegaard M, Dalby Salvig J, Secher NJ (2000). Postpartum depression: identification of women at risk. BJOG.

[9] O'Hara MW, Wisner KL (2014). Perinatal mental illness: definition, description and aetiology. Best Pract Res Clin Obstet Gynaecol.

[10] Robertson E, Grace S, Wallington T, Stewart DE (2004). Antenatal risk factors for postpartum depression: a synthesis of recent literature. Gen Hosp Psychiatry.

[11] Scheele J, Harmsen van der Vliet-Torij HW, Wingelaar-Loomans EM, Goumans M (2020). Defining vulnerability in European pregnant women, a Delphi study. Midwifery..

[12] Brygger Venø L, Jarbøl, DE, Pedersen, LB, Søndergaard, J, Ertmann RK. General practitioners' perceived indicators of vulnerability in pregnancy- A qualitative interview study. BMC Family Practice. 2021 [in press]. 10.1186/s12875-021-01439-3 PMC8236135 34174822

[13] Sundhedsstyrelsen [Danish National Board of Health]. Evaluering af etablering af Familieambulatorierne. Slutevaluering [Evaluation of the Social obstetric outpatient clinics. End evaluation] 2015. https://www.sst.dk/da/udgivelser/2015/evaluering-af-etableringen-af-familieambulatorierne: COWI; 2015.

[14] Khan L (2015). Falling Through the Gaps: Perinatal Mental Health and General Practice.

[15] Buist A, Bilszta J, Milgrom J, Barnett B, Hayes B, Austin MP (2006). Health professional's knowledge and awareness of perinatal depression: results of a national survey. Women Birth.

[16] Ford E, Lee S, Shakespeare J, Ayers S (2017). Diagnosis and management of perinatal depression and anxiety in general practice: a meta-synthesis of qualitative studies. Br J Gen Pract.

[17] Ford E, Shakespeare J, Elias F, Ayers S (2017). Recognition and management of perinatal depression and anxiety by general practitioners: a systematic review. Fam Pract.

[18] Noonan M, Doody O, Jomeen J, O'Regan A, Galvin R (2018). Family physicians perceived role in perinatal mental health: an integrative review. BMC Fam Pract.

[19] Noonan M, Doody O, O'Regan A, Jomeen J, Galvin R (2018). Irish general practitioners' view of perinatal mental health in general practice: a qualitative study. BMC Fam Pract.

[20] Flaherty EG, Sege R (2005). Barriers to physician identification and reporting of child abuse. Pediatr Ann.

[21] Talsma M, Bengtsson Boström K, Östberg AL (2015). Facing suspected child abuse–what keeps Swedish general practitioners from reporting to child protective services?. Scand J Prim Health Care.

[22] Schweitzer RD, Buckley L, Harnett P, Loxton NJ (2006). Predictors of failure by medical practitioners to report suspected child abuse in Queensland, Australia. Aust Health Rev.

[23] Braithwaite J (2018). Changing how we think about healthcare improvement. BMJ.

[24] Brygger Venø L, Bjørnskov Pedersen, L, Søndergaard, J., Ertmann RK, Jarbøl, DE. Assessing and addressing vulnerability in pregnancy. General practitioners perceived barriers and facilitators a qualitative interview study [in press]. BMC Primary Care. 2022. 10.1186/s12875-022-01708-9 PMC9164392 35659201

[25] Tong A, Sainsbury P, Craig J (2007). Consolidated criteria for reporting qualitative research (COREQ): a 32-item checklist for interviews and focus groups. Int J Qual Health Care.

[26] Atkins L, Francis J, Islam R, O'Connor D, Patey A, Ivers N (2017). A guide to using the Theoretical Domains Framework of behaviour change to investigate implementation problems. Implement Sci.

[27] Alexander KE, Brijnath B, Mazza D (2014). Barriers and enablers to delivery of the Healthy Kids Check: an analysis informed by the Theoretical Domains Framework and COM-B model. Implement Sci.

[28] Bar-Zeev Y, Skelton E, Bonevski B, Gruppetta M, Gould GS (2019). Overcoming Challenges to Treating Tobacco use During Pregnancy - A Qualitative study of Australian General Practitioners Barriers. BMC Pregnancy Childbirth.

[29] Blackburn M, Stathi A, Keogh E, Eccleston C (2015). Raising the topic of weight in general practice: perspectives of GPs and primary care nurses. BMJ Open.

[30] Hunter A, Yargawa J, Notley C, Ussher M, Bobak A, Murray RL (2021). Healthcare Professionals' Beliefs, Attitudes, Knowledge, and Behavior Around Vaping in Pregnancy and Postpartum: A Qualitative Study. Nicotine Tob Res.

[31] Mazza D, Chapman A, Michie S (2013). Barriers to the implementation of preconception care guidelines as perceived by general practitioners: a qualitative study. BMC Health Serv Res.

[32] Michie SW, Campbell R, Brown J, Gainforth H (2018). ABC of Behavior Change Theories.

[33] Michie S, Johnston M, Abraham C, Lawton R, Parker D, Walker A (2005). Making psychological theory useful for implementing evidence based practice: a consensus approach. Qual Saf Health Care.

[34] Cane J, O'Connor D, Michie S (2012). Validation of the theoretical domains framework for use in behaviour change and implementation research. Implement Sci.

[35] Michie SA, West L.,R (2014). The Behaviour Change Wheel: A Guide to Designing Interventions.

[36] Krog MD, Nielsen MG, Le JV, Bro F, Christensen KS, Mygind A (2018). Barriers and facilitators to using a web-based tool for diagnosis and monitoring of patients with depression: a qualitative study among Danish general practitioners. BMC Health Serv Res.

[37] McLellan JM, O'Carroll RE, Cheyne H, Dombrowski SU (2019). Investigating midwives' barriers and facilitators to multiple health promotion practice behaviours: a qualitative study using the theoretical domains framework. Implement Sci.

[38] Heslehurst N, Newham J, Maniatopoulos G, Fleetwood C, Robalino S, Rankin J (2014). Implementation of pregnancy weight management and obesity guidelines: a meta-synthesis of healthcare professionals' barriers and facilitators using the Theoretical Domains Framework. Obes Rev.

[39] Pedersen KM, Andersen JS, Søndergaard J (2012). General practice and primary health care in Denmark. J Am Board Fam Med.

[40] Bekendtgørelse af lov om social service § 153 [ministeral order of law of social service](danish). Sect. chapter 27 Duty to report (2019).

[41] danskelove.dk/serviceloven/153. Serviceloven paragraf 153 [The Danish law of social service]

[42] Bekendtgørelse af lov om social service [Ministerial order of law of social serice] (Danish), 1114. Sect. 2018–3828 (2018).

[43] Bekendtgørelse af sundhedsloven [Ministerial order of the Health Legislation] (Danish), (2016).

[44] Databeskyttelsesloven [Danish Data Protection Act ] (Danish). Sect. 2017–7910–0004 (2019).

[45] Malterud K, Siersma VD, Guassora AD (2016). Sample Size in Qualitative Interview Studies: Guided by Information Power. Qual Health Res.

[46] Malterud K (2012). Systematic text condensation: a strategy for qualitative analysis. Scand J Public Health.

[47] Oandasan I, Conn L, Lingard L, Karim A, Jakubovicz D, Whitehead C, et al. The impact of space and time on interprofessional teamwork in Canadian primary health care settings: implications for health care reform. Primary Health Care Research & Development. 2009;10:151-62.

[48] Hetlevik O, Gjesdal S (2010). Norwegian GPs' participation in multidisciplinary meetings: a register-based study from 2007. BMC Health Serv Res.

[49] van den Berg MJ, de Bakker DH, Westert GP, van der Zee J, Groenewegen PP (2009). Do list size and remuneration affect GPs' decisions about how they provide consultations?. BMC Health Serv Res.

[50] Stolper E, Verdenius JP, Dinant GJ, van de Wiel M (2020). GPs' suspicion of child abuse: how does it arise and what is the follow-up?. Scand J Prim Health Care.

[51] Michie S, van Stralen MM, West R. The behaviour change wheel: a new method for characterising and designing behaviour change interventions. Implement Sci. 2011;6:42. 10.1186/1748-5908-6-42 PMC3096582 21513547

