# Supplementary material for: Challenges in the cross-sectoral collaboration on vulnerable pregnant women: a qualitative study among Danish general practitioners
Source: BMC Prim Care. 2022 Jul 26;23:187. doi: 10.1186/s12875-022-01773-0 (PMC9327288; doi:10.1186/s12875-022-01773-0)
Supplement: Supplementary file 1 — Additional file 1: Appendix 1. Interview guide. [file 12875_2022_1773_MOESM1_ESM.pdf]

Interview guide

Character of interview and participants

Focus group interviews with general practitioners (GPs), 3-6 participants (in average 4).

Short introduction by interviewer

Giving a short presentation of the interviewer (LBV) and moderator (DEJ), emphasizing our like-minded position as GPs and part-time researchers, explaining the study's aims, without disclosing our pre-assumptions.

Overall aim: exploring the GPs experience working with vulnerable pregnant women. Explaining the partial aims.

Instructing the participants: the interview is meant to be an informal discussion, only partially structured. Ensuring that all experience and attitudes are acceptable and of equal importance, that there is no right or wrong, as there are no clear definitions of vulnerable pregnant women. Encouraging participants to share experience of things that were difficult or even went wrong.

Ensuring ethics: anonymous forum, data protection rules, duty of confidentiality

Gaining written consent from participants

Themes	Questions
Presentation of all participants	Practice location, practice type, seniority, average number of pregnancy consultations per month
Conceptualizing vulnerability in pregnancy	Think of a pregnancy consultation, e.g. the first pregnancy consultation, with a vulnerable woman who made you worry how things would go – define what made you worry. Also, in your own words, define who you consider to be vulnerable pregnant women. Welcoming clinical examples.
Organization of antenatal care	How do you organize antenatal care consultations in your clinic?
Assessing and addressing vulnerability	How do you, among all other tasks in antenatal care consultations, focus on assessing the vulnerability of the pregnant women?<br>How do you get the suspicion that a pregnant woman might be vulnerable?<br>What might prevent you from thinking about a pregnant woman's vulnerability?<br>When clarifying signs of vulnerability, what do you find important to assess in the patient's medical history?<br>How do you address vulnerability to the pregnant woman and her partner? And what can be difficult to address?
The cross-sectoral collaboration	How do you handle the obtained information about indicators of vulnerability?<br>What do you generally document in the pregnancy chart?<br>Do you typically refer the woman to collaborative partners? Which partners?<br>How do you perceive the cross-sectoral antenatal care collaboration?<br>How do you perceive the task of navigating in the cross-sectoral collaboration?<br>How do you perceive the task of communicating with the cross-sectoral partners? (the social-obstetric units, specialists in the secondary sector, municipal health visitors, municipal social authorities)

	Have you ever made a report to the social authorities about a vulnerable pregnant woman?<br>In which situation would you find it necessary to report a vulnerable pregnant woman?<br>Is it difficult to report vulnerable pregnant women? Why? What are the challenges?
Wished changes	What changes might make it easier for you to assess vulnerability among pregnant women?<br>Do you have wishes for changes in the cross-sectoral collaboration of antenatal care which would ease assessing vulnerable pregnant women?<br>Do you have wishes for changes in the collective agreement - which would ease assessing vulnerable pregnant women?
Round-off	Thanking for participation
