# Supplementary material for: Challenges in the cross-sectoral collaboration on vulnerable pregnant women: a qualitative study among Danish general practitioners
Source: BMC Prim Care. 2022 Jul 26;23:187. doi: 10.1186/s12875-022-01773-0 (PMC9327288; doi:10.1186/s12875-022-01773-0)
Supplement: Supplementary file 2 — Additional file 2: Appendix 2. COREQ items. [file 12875_2022_1773_MOESM2_ESM.pdf]

COREQ items

Domain 1: Research team and reflexivity

Personal Characteristics

1. Interviewer/facilitator: The first author LBV conducted the interviews with DEJ as an experienced moderator
2. Credentials: The main author LBV is a GP and Ph.D. student at the Research Unit of General Medicine at University of Southern Denmark. DEJ and JS are part time GPs and professors at the Research Unit of General Medicine at University of Southern Denmark. RE is a part time GP and associate professor at the research Unit of general medicine, University of Copenhagen. LIB is a health economists and associate professor at the Research Unit of General Medicine at University of Southern Denmark.
3. Occupation: LBV work as a part time GP and part time Ph.D. student
4. Gender: LBV is female
5. Experience and training: LBV have attended Ph.D. courses in qualitative study designs and writing qualitative articles. DEJ, JS and RE are all senior researchers with experience of qualitative and quantitative research traditions. LIB is a senior researcher with experience in survey studies

Relationship with participants

6. Relationship established: The first interview was a pilot with GPs (n=5) working as part time researchers in our research unit, and therefore had a prior knowledge to the researcher team.
7. Participant knowledge of the interviewer: Prior to interviews the interviewer presented the study aim but without presenting our prior experiences and pre-assumptions in the field
8. Interviewer characteristics: The interviewer acted friendly, and like-minded among participants. The interview guide provided a flexible frame with open-ended questions about the GPs' perceived barriers when collaborating and reporting on vulnerability among pregnant women and welcoming clinical examples. Ongoing adjustments of the interview guide were made to elaborate on new perceptions.

Domain 2: study design

Theoretical framework

9. Methodological orientation and theory: We chose the qualitative methodology to explore GPs' perceived barriers and facilitators when collaborating in ANC and reporting on vulnerability in pregnant women. A qualitative design enabled us to explore the GPs' perceived barriers and facilitators in the terms of "what, why, and how". The safe environment during the interview encouraged them to disclose situations of deficient performance when collaborating or reporting on vulnerable pregnant women, and the dialogue rendered the GPs to reflect on their own practices.
   We applied a pragmatic clinical empirical approach not driven by prior established theoretical frameworks or research traditions, as elaborated by Malterud((1) p. 124). However, recognizing that our stance is always affected by theory, during process of analysis we searched for theories to support our data interpretation. In this study we chose the Theoretical Domains Framework(2) as a theoretic model to understand the GPs' behavior and perceived barriers and facilitators when collaborating and reporting on vulnerable pregnant women.

Participant selection

10. Sampling: Reported in the Data collection section, p. 8, line 167-171.
11. Method of approach: Reported in the Data collection section, p. 8, line 172-173
12. Sample size: Due to slow recruitment, the end sample consisted of a convenience sample of twenty GPs representing only partnership practices
13. Non-participation: Almost 60 GPs were contacted, and main reasons for decline was a high workload.

Setting

14. Setting of data collection: The interviews took place at the research unit of general practice in Odense or in the local practice area of participating GPs.

15. Presence of non-participants: No other besides participants, the interviewer LBV and moderator DEJ was present
16. Description of sample: Reported in table 1.

Data collection

17. Interview guide: The interview guide provided a flexible frame with open-ended questions about the GPs' perceptions of enablers and barriers in collaboration and reporting on vulnerable pregnant women and welcoming clinical examples. Ongoing adjustments of the interview guide were made to elaborate on new perceptions.
18. Repeat interviews: five focus group discussion were made, which included the pilot test
19. Audio/visual recording: All interviews were audio recorded and transcribed verbatim by LBV
20. Field notes: the author made field notes after almost all interviews about the characteristics of the interview
21. Duration: each interview lasted approximately 60 minutes
22. Data saturation: data saturation was discussed among authors
23. Transcripts returned: No transcripts were returned to participants for comments or corrections.

Domain 3: analysis and findings

Data analysis:

24. Number of data coders: All authors read the first two interviews. After the themes were discussed among the authors, LBV conducted the initial inductive coding. The following stepwise inductive analysis was conducted by LBV in cooperation with RE. During the process of inductive analysis, we chose to apply TDF as a theoretic model to guide the deductive analysis. LBV conducted the deductive coding to the domains of TDF, assisted by JVL, who is a MD, Ph.D. with experience in behavioral research. The research team discussed and reflected on the findings until consensus was reached.
25. Description of the coding tree: The deductive coding is illustrated in table 1.
26. Derivation of themes: The three themes: (I)Collaborative experience, (II) Motivation, (III)Organizational working conditions, were identified from the data in both the inductive and deductive analysis.
27. Software: NVivo pro version 12 was used to organize the data
28. Participant checking: As no transcripts was returned to participants, no participants provided feedback.

Reporting

29. Quotations presented: quotations were presented to illustrate themes, identified by participant number.
30. Data and findings consistent: Consistency were found between the data and the findings, as the findings were recontextualized against the origin interview material.
31. Clarity of major themes: The three major themes covering twelve TDF domains were clearly presented in the findings.
32. Clarity of minor themes: categories of minor themes were presented in the text and table 3.

1. Malterud K. Theory and interpretation in qualitative studies from general practice: Why and how? Scand J Public Health. 2016;44(2):120-9.
2. Cane J, O'Connor D, Michie S. Validation of the theoretical domains framework for use in behaviour change and implementation research. Implement Sci. 2012;7:37.
