# Supplementary material for: Challenges in the cross-sectoral collaboration on vulnerable pregnant women: a qualitative study among Danish general practitioners
Source: BMC Prim Care. 2022 Jul 26;23:187. doi: 10.1186/s12875-022-01773-0 (PMC9327288; doi:10.1186/s12875-022-01773-0)
Supplement: Supplementary file 3 — Additional file 3: Appendix 3. Theoretical Domains Framework (TDF), domains and constructs coding manual. [file 12875_2022_1773_MOESM3_ESM.pdf]

Appendix: Theoretical Domains Framework (TDF), domains and constructs coding manual

Adapted from Michie et al 2005 and Cane et al 2012

	**Domains and belonging constructs**<br>The domains are registered by number<br>Italic text refers to constructs adapted from Michie 2005 and Cane 2012	**Examples from the relevant data**<br>Abbreviations:<br>ANC = antenatal care<br>HCPs = health care professionals
COM-B: psychological CAPABILITY	**Knowledge (1)**<br>• Knowledge of condition/scientific rationale<br>• Procedural knowledge (how to do it)<br>• Schemas + mindsets + illness representations	• Statements about having/not having /wanting **factual knowledge** of the condition and the rationale behind doing the behaviour.<br>• Statements about having/not having /wanting **procedural knowledge** of when and how to do the behavior<br>• Statements about having/not having /wanting **rationale** of doing the behaviour<br>In the context of this study, knowledge could relate to factual knowledge about the risks of vulnerability in pregnancy and the risk for the child. Procedural knowledge could relate to knowledge of care pathways in different levels of ANC for vulnerable pregnant women, and about when to report. Rationale could relate to knowledge about obliged duty to report, which cannot be delegated to other HCPs. May be both correct and incorrect knowledge.
COM-B: physical and psychological CAPABILITY	**Skills (2)**<br>• Skills Development *(the gradual advancement through progressive stages of an ability or proficiency acquired through training and practice)*<br>• Competence (*one's repertoire of skills, and ability especially as it is applied to a task or set of tasks)*<br>• Ability *(competence or capacity to perform a physical or mental act. Ability may be either unlearned or acquired by education and practice)*	• Statements describing techniques/capability/skills used/how they do the behaviour<br>• Statements of wanting to develop/improve the skills doing the behavior<br>In the context of this study, skills may be interpersonal skills (e.g. using empathy, sensitivity, practical advice, promote benefits, non-judgmental approach, terminology, normalization, communication skills etc.) in relation to cross-sectoral collaboration and reporting on vulnerable pregnant women. May also be skills adopted to cope in the absence of specific skills training related to reporting.

	• Interpersonal skills *(an aptitude enabling a person to carry on effective relationships with others, such as an ability to cooperate, to assume appropriate social responsibilities or to exhibit adequate flexibility)*<br>• Practice *(repetition of an act, behavior or series of activities, of the to improve performance or acquire a skill)*<br>• *Skill assessment (a judgement of the quality, worth, importance, level or value of an ability or proficiency acquired through training and practice)*<br>• Coping strategies	
COM-B: psychological CAPABILITY	**Memory, attention, and decision processes (10)**<br>• Memory: (*ability to retain information or representation of a past experience based on the mental processes of learning)*<br>• Attention: *(awareness, senses are focused selectively on aspects of the environment, readiness to respond to stimuli)*<br>• Attention control: *(concentration on relevant cues and ignore irrelevant cues in a given situation)*<br>• Decision making*: (cognitive process of choosing between two or more alternatives)*	• Statements relating to time/situations etc. when the GPs would remember/forget to do the behavior.<br>• Statements relating to relying on cognitive approaches to do the behaviour<br>• Statements relating to cognitive limitations such as forgetting/overseeing/not being able to decide whether to do the behaviour<br>In the context of this study, memory, attention, and decision processes may relate to the GPs ability to remember to engage in the cross-sectoral collaboration in ANC or to report when necessary. Approaches to collaborating and reporting. Decision processes may relate to the GPs decision when or whether to report, or delegating the decision to other HCPs, despite the fact the duty of reporting is personal
COM-B: psychological CAPABILIY	**Behavioral regulation (14)**<br>• Self-monitoring *(keeping records of behavior in connection with efforts to change or regulate, the ability to modify one's behavior in response to a situation)*<br>• Action planning<br>• Project management	• Statements where GPs wants audit/evaluation/feedback on their behavior<br>• Statements about prompts or processes used to make the behaviour sustainable<br>• Statements about efforts to ensure doing the behaviour<br>In the context of this study behavioral regulation may relate to wishes of feedback on collaboration/reporting, or local prompts used to improve the collaborating in ANC or reporting on pregnant women.

COM-B: reflective MOTIVATION	**Social and professional role and identity (self-standards) (3)**<br>• Professional identity *(The characteristics by which an individual is recognized relating to, connected with or befitting a particular profession)*<br>• Professional role *(The behavior considered appropriate for a particular kind of work or social position)*<br>• Social identity *(The set of behavioral or personal characteristics by which an individual is recognizable [and portrays] as a member of a social group)*<br>• Identity *(An individual's sense of self defined by a) a set of physical and psychological characteristics that is not wholly shared with any other person and b) a range of social and interpersonal affiliations (e.g., ethnicity) and social roles)*<br>• Professional boundaries *(The bounds or limits relating to, or connected with a profession or calling)*<br>• Professional confidence *(An individual's belief in his or her repertoire of skills, and ability especially as it is applied to a task or set of tasks)*<br>• Group identity *(The set of behavioral or personal characteristics by which an individual is recognizable [and portrays] as a member of a group)*<br>• Leadership *(The processes involved in leading others, including organizing, directing,*	• Statements relating to how GPs' sees themselves<br>• Statements relating to the extent they view the behavior as a characteristic/meaningful task representative of their professional role<br>• Statements relating to the extent their personal identity influences the behavior<br>○ Part of our role/job/responsibility to do the behavior<br>○ My personal identity impacts on how I do the behavior<br><u>In the context of this study</u>, professional role may relate to the extent that GPs' feels that collaborating and reporting about vulnerable pregnant women is part of their professional role. Furthermore, it may relate professional challenges in collaboration and reporting. Personal identity may relate to the GPs gender or status as being a parent and the impact this has on engaging in cross-sectoral collaboration and reporting on vulnerable pregnant women

	coordinating and motivating their efforts toward achievement of certain group or organization goals)<br>• Organizational commitment (*emotional or moral element, prudent element – a dedication to an organization wishing to remain part of it)*	
COM-B: reflective MOTIVATION	**Beliefs about capability (self-efficacy) (4)**<br>• Self-confidence *(trust in one's own abilities, capabilities, and judgement)*<br>• Perceived competence (*belief in his/her ability to learn and execute skills)*<br>• Perceived behavioral control *(perception of the ease or difficulty of performing the behavior of interest)*<br>• Self-esteem (*the degree to which the qualities and characteristics contained in one's self-concept are perceived to be positive)*<br>• Empowerment (*the promotion of the skills, knowledge, and confidence necessary to take great control of one's life; as in certain educational or social schemes; the delegation of increased decision-making powers to individuals or groups in a society or organization)*<br>• Professional confidence (*an individual's belief in his/her repertoire of skills, and ability – especially as it is applicated to a task or set of tasks)*<br>• Control of behavior and material and social environment	• Evaluative statements about GPs' confidence and judgement about their competence and control in their ability or inability to do the behavior<br>○ I feel that I have/don't have control doing the behavior<br>• Statements relating to expectations of carrying out the behavior due to beliefs of own competencies.<br>○ I know that doing the behavior won't be successful because I'm/I'm not very efficient at doing this task<br><u>In the context of this study,</u> beliefs about capability relates to the GPs making evaluative judgements on their ability to collaborate in ANC or report on vulnerable pregnant women, e.g., their confidence in being able to have an overview of the ANC for vulnerable pregnant women- or to decide the necessity of reporting on vulnerable pregnant women.
COM-B: reflective MOTIVATION	**Optimism (5)**<br>• Optimism: *the confidence that things will happen for the best or that desired goals will be attained)*	• Optimism or pessimism –confidence that the problem can/can't be solved.<br><u>In the context of this study,</u> this would include expressing optimism/pessimism of the effects of referring for social-obstetric

	• Pessimism (*the attitude that things will go wrong and that peoples wishes or aims are unlikely to be fulfilled)*	specialized care, engaging in the cross-sectoral ANC, and reporting to the social services in the municipality
COM-B: reflective MOTIVATION	**Beliefs about consequences (anticipated outcomes/attitude) (6)**<br>• Beliefs *(The thing believed; the proposition or set of propositions held true)*<br>• Outcome expectancies *(cognitive, emotional, behavioral and affective outcomes that are assumed to be associated with future or intended behaviors. These assumed outcomes can either promote or inhibit future behaviors)*<br>• Characteristics of outcome *expectancies (characteristics of the cognitive, emotional and behavioral outcomes that individuals believe are associated with future or intended behaviors and that are believed to either promote or inhibit these behaviors. These include whether they are sanctions/rewards, probable/improbable, perceived risk or threats)*<br>• Anticipated regret (*a sense of the potential negative consequences of a decision that influences the choice made)*<br>• Consequents (*an outcome of behavior in a given situation)*	• Statements relating to GPs beliefs about the outcome/consequences of doing the behavior<br>• Statements can include consequences of on their patient<br>○ If I do/don't do …. X, y, z will happen<br>○ Doing the behavior will have a beneficial /adverse impact on my patient<br>○ Doing/not doing the behavior, positive or negative consequences of the behavior<br><u>In the context of this study,</u> beliefs about consequences could relate to GPs beliefs that the outcome/consequences of engaging in the cross-sectoral collaboration – or reporting about vulnerable pregnant women will result consequences on the GP-patient interaction
COM-B: Reflective MOTIVATION	**Intentions (8)**<br>A conscious decision to perform a behavior in a certain way<br>• Stability of intentions: (ability of one's resolve to remain despite disturbing influences)<br>• Stages of change model<br>• Trans theoretical model and stages of change: (a five-stage theory to explain changes in health behavior. It suggests that change takes time, that	• Things I want to do<br>• Statements relating to the extent that GPs planning to engage in the behavior.<br><br><u>In the context of this study,</u> intentions may be GPs' stating how they aim to collaborate by engaging in the cross-sectoral ANC collaboration when seeing a pregnant woman – or aim to increase focus on reporting. Conversely weak intentions may be a lack of intention to engage in

	different interventions are effective at different stages, and that there are multiple outcomes occurring across stages)<br>• Intrinsic motivation<br>• Commitments	collaboration or reporting on vulnerable pregnant women (e.g., I don't always make a point of ….)
COM-B: Reflective MOTIVATION	**Goals (9)**<br>Mental representations of outcomes or end states that an individual wants to achieve<br>• Goals: desired state of affairs of a system or person, may be proximal (closer) or distal (further away)<br>• Goal priority<br>• Goal target setting specific time-based behavior targets that are measurable, achievable, and realistic	• Things I want to achieve<br>• Statements relating to GPs goals/aims/desired result of doing the behavior (ex. reporting to authorities),<br>• Statements relating to how prioritizing goals influences whether to do the behavior<br>○ competing priorities mean I don't do the behavior<br>○ I prioritize other behaviors which are more important<br>In the context of this study, GPs goals may relate to wanting to support vulnerable pregnant women during their pregnancy to get the right supportive care, their family's health, and pregnancy outcomes, to reduce risks. Goal priorities may relate to the competing task in general practice – i.e., other patient categories or administrative tasks. How important ANC for vulnerable pregnant women is perceived in relation to other priorities of primary care.
COM-B: automatic MOTIVATION	**Reinforcement (7)**<br>• Rewards *(proximal / distal, valued / not valued, probable /improbable) (Return or recompense made to, or received by a person contingent on some performance)*<br>• Incentives (*an external condition that enhances or serves as a motive for behavior*)<br>• Punishment (*an unwanted or undesired event imposed as a penalty on a wrongdoer*)<br>• Consequents (*an outcome of behavior in a given situation*)<br>• Reinforcement (Increasing the probability of a response by arranging a dependent relationship or contingency between the response and a given stimulus)	• Statements relating collaborating or reporting being directly contingents on receiving rewards or punishments<br>○ Otherwise, x, y, z will /will not happen<br>○ Getting thanked for encourages me to do….<br>○ I will get in trouble/be reported if I don't…<br><br>In the context of this study, reinforcement could be related to the incentives that motivates for engaging in the cross-sectoral ANC or reporting on vulnerable pregnant women.

	• Contingencies *(A conditional probabilistic relation between two events. Contingencies may be arranged via dependencies or they may emerge by accident)*<br>• Sanctions (*A punishment or other coercive measure, usually administered by a recognized authority, that is used to penalize and deter inappropriate or unauthorized actions*	
COM-B: Automatic MOTIVATION	**Emotion (13)**<br>• Fear *(An intense emotion aroused by the detection of imminent threat, involving an immediate alarm reaction that mobilizes the organism by triggering a set of physiological changes)*<br>• Anxiety *(A mood state characterized by apprehension and somatic symptoms of tension in which an individual anticipates impending danger, catastrophe, or misfortune)*<br>• Affect *(An experience or feeling of emotion, ranging from suffering to elation, from the simplest to the most complex sensations of feelings, and from the most normal to the most pathological emotional reactions)*<br>• Stress *(A state of physiological or psychological response to internal or external stressors)*<br>• Depression *(A mental state that presents with depressed mood, loss of interest or pleasure, feelings of guilt or low self-worth, disturbed sleep or appetite, low energy, and poor concentration)*<br>• Positive / negative affect *(The internal feeling/state that occurs when a goal has/has not been attained, a source of threat has/has not been avoided, or the individual is/is not satisfied with the present state of affairs)*<br>• Burn-out *(Physical, emotional, or mental exhaustion, especially in one's job or career,*	• An expression of GP own personal emotional reaction,<br>• Expression how this positively/negatively impact on them engaging in doing the behavior, e.g. fear/anxiety, affect, stress, depression, positive/negative affect, burn-out.<br>In the context of this study, how this positively/negatively impact on them engaging in collaboration/reporting on vulnerable pregnant women, e.g. causing positive/negative affect, stress, burn-out.<br>Emotion relates to the emotional responses of the GPs, and not the emotional responses of the patients in relation to collaborating/reporting on vulnerability in a pregnant woman.

	accompanied by decreased motivation, lowered performance and negative attitudes towards oneself and others)<br>• Cognitive overload / tiredness *(The situation in which the demands placed on a person by mental work are greater than a person's mental abilities)*	
COM-B: physical OPPORTUNITY	**Environmental context and resources (11)**<br>• Environmental stressors *(external factors in the environment that cause stress)*<br>• Resources/material resources (*availability and management- human resources used in enacting a behavior)*<br>• Organizational culture/climate (*a distinctive pattern of thought and behavior shared by members of the same organization and reflected in their language, values, attitudes, beliefs and customs)*<br>• Critical incidents *(occurrences that one judges to be distinctive, prominent or otherwise significant)*<br>• Person x environment interaction (*interplay between the individual and their surroundings)*<br>• Knowledge of task environment (*knowledge of the social and material context in which a task is undertaken)*	• Statements describing the presence/absence of tools/resources/equipment/services/organizational structures which facilitates/limits the GPs in doing the behavior.<br>• Wished tools/resources/services/changes in the organizational structure to facilitate the behavior<br>In the context of this study, examples on the environmental contest and resources could be the availability of support services, service-level pathways of care for vulnerable pregnant women, whether organization culture prioritizes/provides the necessary information on vulnerable pregnant women (NOTE: in relation to having to priorities behaviors due to time restrictions then time would be coded as goals).
COM-B: social OPPORTUNITY	**Social influences (12)** (*those interpersonal processes that can cause individuals to change their thoughts, feelings or behaviors)*<br>• Social pressure *(the exertion of influence on a person or group by another person or group)*<br>• Social norms (*socially determined consensual standards)*	• Statements expressing the influence of others on doing/not doing the behavior (social support, group norms etc.)<br>○ I do/don't do the behavior because others support/advocate/disapprove/dictate/demand it<br>In the context of this study, the influence from others may include individuals or groups of peers (other GPs), cross-sectoral collaborators (hospital specialist doctors, midwifes, nurses, health-visitors, social workers from the municipality) or authoritative organizations etc.

	• Group conformity *(the act of consciously maintaining a certain degree of similarity to those in your general social circles)*<br>• Social comparisons (*the process by which people evaluate their attitudes, abilities or performance relative to others*)<br>• Group norms *(any behavior, belief, attitude or emotional reaction held to be correct or acceptable by a given group in society)*<br>• Social support<br>• Power<br>• Intergroup conflict<br>• Alienation (deep sense of dissatisfaction with one's personal experiences that can be a source of lack of trust in one's social or physical environment or in oneself)<br>• Group identity (*set of behavioral or personal characteristic by which an individual is recognizable and portrays as a member of a group)*<br>• Modelling<br>• Organizational culture/climate *(a distinctive pattern of thought and behavior shared by members of the same organization and reflected in their language, values, attitudes, beliefs and customs)*	(NOTE: not the interpersonal behavior of collaborating or reporting = skills)
